# Techniques to mitigate lead migration for percutaneous trials of cervical spinal cord stimulation

**DOI:** 10.3389/fsurg.2025.1458572

**Published:** 2025-03-31

**Authors:** Jonathan N. Finney, Isaiah R. Levy, Santosh Chandrasekaran, Jennifer L. Collinger, Michael L. Boninger, Robert A. Gaunt, Eric R. Helm, Lee E. Fisher

**Affiliations:** ^1^ Department of Physical Medicine and Rehabilitation, University of Pittsburgh, Pittsburgh, PA, United States; ^2^ Rehab Neural Engineering Labs, University of Pittsburgh, Pittsburgh, PA, United States; ^3^ Neural Bypass and Brain Computer Interface Lab, Feinstein Institutes for Medical Research, Northwell Health, Manhasset, NY, United States; ^4^ Department of Bioengineering, University of Pittsburgh, Pittsburgh, PA, United States; ^5^ Center for Neural Basis of Cognition, Pittsburgh, PA, United States; ^6^ Department of Biomedical Engineering, Carnegie Mellon University, Pittsburgh, PA, United States

**Keywords:** neuropathic pain, spinal cord stimulation, lead migration, neuroprosthetics, cervical spine

## Abstract

**Introduction:**

Epidural spinal cord stimulation (SCS) is a clinical neuromodulation technique that is commonly used to treat neuropathic pain, with patients typically undergoing a one-week percutaneous trial phase before permanent implantation. Traditional SCS involves stimulation of the thoracic spinal cord, but there has been substantial recent interest in cervical SCS to treat upper extremity pain, restore sensation from the missing hand after amputation, or restore motor function to paretic limbs in people with stroke and spinal cord injury. Because of the additional mobility of the neck, as compared to the trunk, lead migration can be a major challenge for cervical SCS, especially during the percutaneous trial phase. The objective of this study was to optimize the implantation procedure of cervical SCS leads to minimize lead migration and increase lead stability during SCS trials.

**Methods:**

In this study, four subjects underwent percutaneous placement of three SCS leads targeting the cervical spinal segments as part of a clinical trial aiming to restore sensation for people with upper-limb amputation. The leads were maintained for up to 29 days and weekly x-ray imaging was used to measure rostrocaudal and mediolateral lead migration based on bony landmarks.

**Results and discussion:**

Lead migration was primarily confined to the rostrocaudal axis with the most migration occurring during the first week. Iterative improvements to the implantation procedure were implemented to increase lead stability across subjects. There was a decrease in lead migration for patients who had more rostral placement of the SCS leads. The average migration from the day of lead implant to lead removal was 29.7 mm for Subject 1 (lead placement ranging from T3-T4 to T1-T2), 41.9 mm for Subject 2 (T2-T3 to C7-T1), 1.9 mm for Subject 3 (T1-T2 to C7-T1), and 16.6 mm for Subject 4 (T1-T2 to C7-T1). We found that initial placement of spinal cord stimulator leads in the cervical epidural space as rostral as possible was critical to minimizing subsequent rostrocaudal lead migration.

## 1 Introduction

Pain conditions in the distal limbs, such as peripheral neuropathy, phantom limb pain, and complex regional pain syndrome, are difficult to treat. Spinal cord stimulation (SCS) is often effective in reducing pain in the proximal limbs and trunk, but the technique has been less successful for distal limb pain (1). Traditionally, SCS involves electrical stimulation of the dorsal columns to activate Aβ neurons, which act as a gate for pain pathways (2). With this technique, pain relief occurs when stimulation evokes a paresthetic sensation at the same location as the pain and has demonstrated successful relief of chronic neuropathic pain with stimulation of the cervical spinal cord (3–6). In addition to pain relief, cervical SCS can be useful for functional restoration such as improving motor function after stroke (7). However, with traditional SCS, it is often challenging or impossible to generate focal paresthetic coverage of distal pain (and associated pain relief) without generating extraneous paresthesia in uninvolved off-target regions of the body (8). As such, multiple studies have targeted the lateral spinal cord, near the dorsal rootlets and dorsal root entry zone to selectively stimulation primary afferents where they enter the cord (7,9,10).

An additional challenge of SCS is lead migration which is a common clinical complication, especially during the percutaneous trial phase that precedes permanent implantation (8). Migration, which describes both rostral-caudal and/or mediolateral movement in the epidural space, is clinically relevant because it may change the region of stimulation, potentially decreasing effectiveness for pain relief and/or functional restoration if the leads are too distant from target sites. Lead migration in SCS is a commonly encountered challenge faced by clinicians, with an incidence reported as high as 88.5% with the vast majority of migration in the caudal direction (1,11,12). A recent retrospective review on percutaneous SCS lead migration cited a substantially lower rate of lead migration requiring revision (2.1%) (13). As the review notes, this low incidence reflects the revision rate (i.e., in which the lead migration was substantial enough to merit revision surgery) rather than the true lead migration rate (i.e., lead movement which may change the region of activation but can be managed by retuning stimulation parameters).

**Figure 1:**
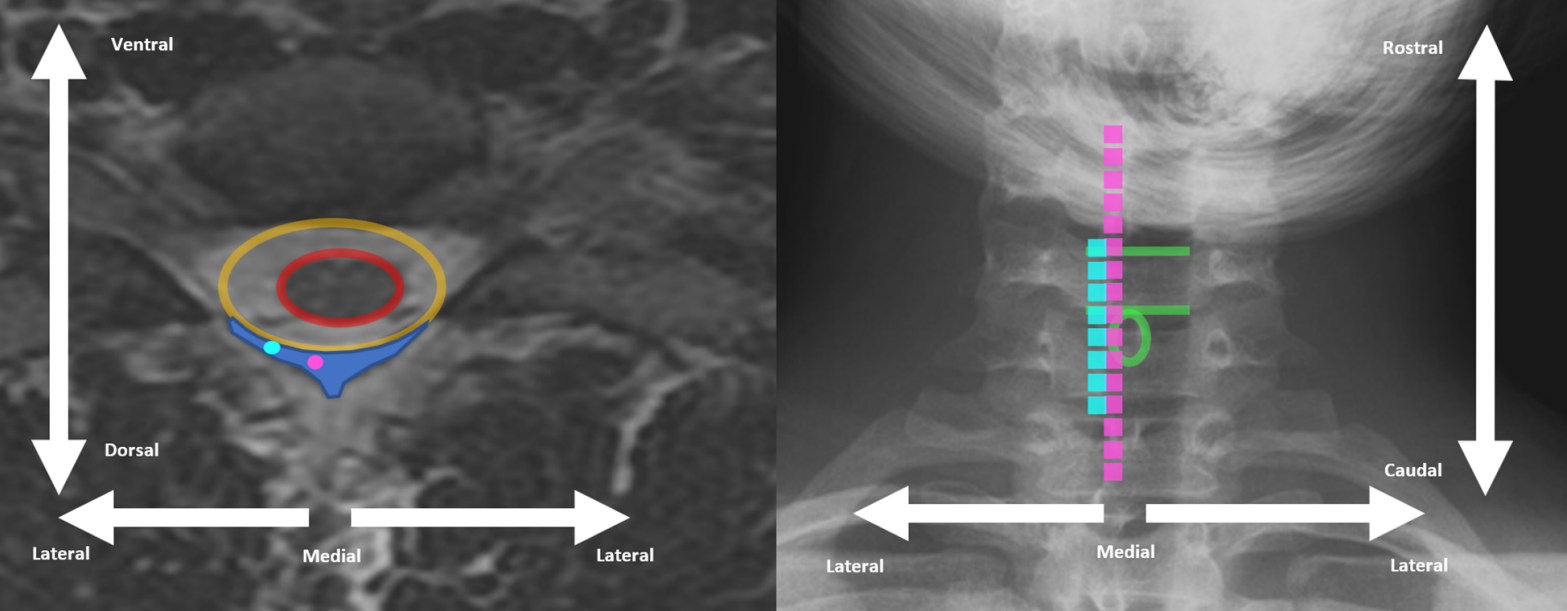
Example cervical spine MRI axial view (left) and cervical spine x-ray (right) demonstrating differences between traditional dorsal column SCS placement (pink) and more lateral placement (light blue). Spinal cord is encircled in red, dural space is encircled in orange, and epidural space is indicated by dark blue shading on left. Superior and inferior end-plate of example vertebral body indicated with green line on right. Spinous process of same vertebral level encircles in green circle on right.

Recently, we have begun to explore stimulation of the lateral cervical spinal cord (Figure 1) as a means to evoke focal sensations in the distal upper extremities as part of a project to restore meaningful sensory feedback from the hand and reduce phantom limb pain after upper-limb amputation (14,15). Targeting the lateral spinal cord was, in part, motivated by some of the success seen with dorsal root ganglion stimulation (DRGS) in treating pain in the distal extremities. DRGS has emerged as a novel neuromodulatory technique to address some of the limitations of SCS and has shown promise in the ability to generate focal coverage of distal limb pain while also reducing the occurrence of lead migration (16–18). DRGS, however, has not received FDA approval for use in the cervical spine. Additionally, DRGS is a technically demanding procedure that requires additional specialized training for the operator and carries increased risk of complications such as lead fracture (18).

Here, we report on the techniques we iteratively developed to improve the approach to percutaneous implantation of SCS leads to reduce migration and increase stability of the cervical SCS leads. By improving stability of lateral cervical SCS lead placement, our goal is to maintain both pain relief and functional restoration facilitated by SCS.

## 2 Materials and methods

Four subjects (Table 1) with upper-limb amputations were implanted with cervical SCS leads as part of a study seeking to restore sensation from a sensorized prosthetic limb and characterize modulation of phantom limb pain (PLP) (14). Subject ages ranged from 33 to 67 years at time of trial. Subjects were assessed anywhere from 2 to 16 years following initial amputation and included both traumatic and non-traumatic etiologies for amputation. All study procedures were approved by the University of Pittsburgh and Army Research Labs Institutional Review Boards and subjects provided informed consent before participation.

**Table 1:** Subject characteristics.

Participant	Age	Sex	Type of amputation	Cause of amputation
Subject 1	67 years	Female	Right shoulder disarticulation	Necrotizing fasciitis
Subject 2	33 years	Male	Left transhumeral	Trauma
Subject 3	38 years	Female	Right transhumeral	Compartment syndrome
Subject 4	44 year	Female	Right transradial	Compartment syndrome

Each subject underwent percutaneous placement of three SCS leads targeting the lateral C5-C8 spinal segments. All procedures were performed by the same physician (author ER Helm). For each lead insertion, the targeted interlaminar space was identified, local anesthetic was administered, and the desired epidural space was accessed via a 14-gauge 4-inch epidural Tuohy needle. Subsequently, the physician percutaneously advanced three 8- or 16-contact SCS leads (Infinion, Boston Scientific) to the lateral epidural space of the C5-C8 spinal segments, with use of a stylet for steering and live fluoroscopy for guidance. Contacts were 3 mm long with 1 mm spaces between contacts.

Although the same physician performed each lead placement, different procedural approaches were used across subjects with 8- or 16-contact leads placement ranging between the C7-T1 and T3-T4 spaces (Table 2). The location of the electrodes was adjusted intraoperatively based on each subject's report of the sensations evoked by stimulation. Once lead placement was deemed adequate for evoking sensations in the missing hand, the physician removed each stylet followed by each needle. Leads were sutured to the skin near the lead exit site using anchors (Boston Scientific), and then covered with sterile dressings. The duration of each procedure was approximately 3–4 h. The leads were maintained for up to 29 days and, at the end of the study, all sutures were removed and leads were explanted with gentle traction on the external portion of the lead.

**Table 2:** Interlaminar space used to gain epidural access.

Participant	First lead	Second lead	Third lead
Subject 1	T3-T4	T2-T3	T1-T2
Subject 2	T1-T2	T2-T3	C7-T1
Subject 3	C7-T1	T1-T2	C7-T1
Subject 4	T1-T2	C7-T1	C7-T1

**Figure 2:**
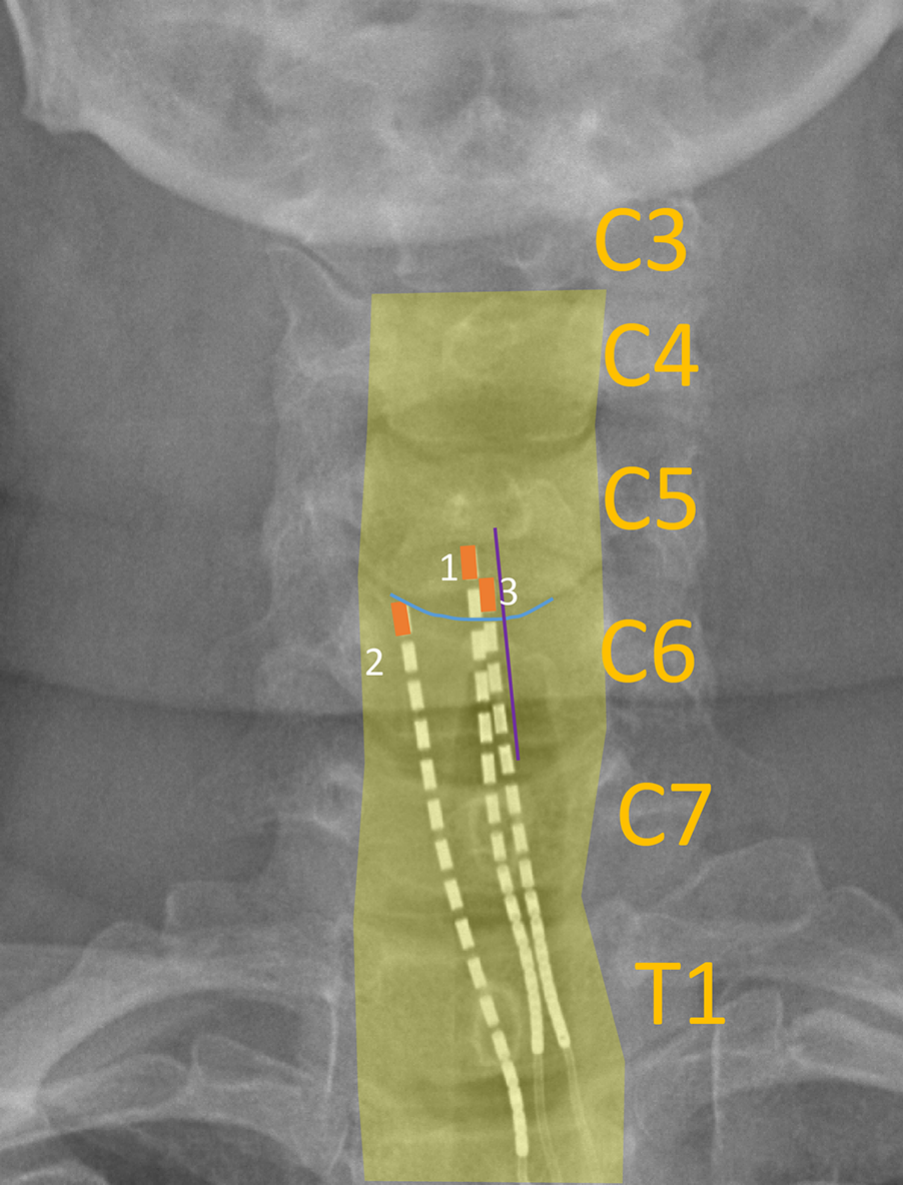
Example x-ray image labelled with landmarks used to calculate lead migration across weeks. Yellow bordering: outline of vertebral bodies; blue line: end plate (superior end plate of C6); purple line: bisection of spinous process (C6); orange box: outline of most rostral SCS contact on each lead.

To quantify lead migration, weekly anteroposterior and lateral x-rays were collected and compared to the intraoperative fluoroscopic images which were collected after removal of all Tuohy needles and stylets. To characterize lead migration from one week to the next, we used bony landmarks to precisely measure the difference in lead location on successive x-ray images. For example, rostrocaudal migration was measured based on the vertebral body end-plate that was most consistently parallel with the fluoroscopic beam (i.e., “squared off”) and mediolateral migration was measured based on the spinous process that was most consistently midline as gauged by its appearance being centered between the pedicles of the corresponding vertebral level. Measurements were taken from x-rays obtained across all weeks (example shown in Figure 2, all images in Supplementary Figures 1–S4). Landmarks that were used for each subject are shown in Table 3. X-ray image measurements were calibrated based on the size of the 3 mm SCS contacts in the image.

**Table 3:** Reference anatomic measurements by which lead migration was measured.

Participant	Rostrocaudal	Lateral
Subject 1	T1 SEP	T1 SP
Subject 2	C5 SEP	C5 SP
Subject 3	C6 SEP	C6 SP
Subject 4	C3 IEP	C4 SP

SEP, superior end plate; IEP, inferior end plate; SP, spinous process.

## 3 Results

**Figure 3:**
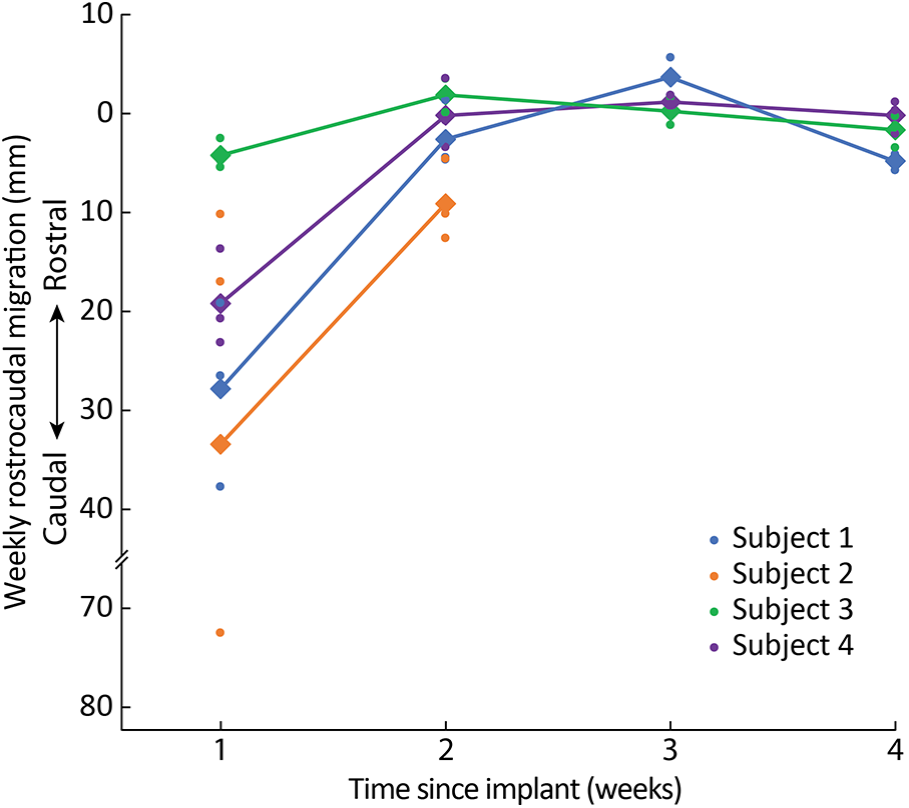
Rostrocaudal migration across weeks. Dots represent the migration of individual leads and diamonds/lines represent the average migration across all leads for each subject.

When comparing the intraoperative fluoroscopy image to all subsequent images, we found that lead migration was primarily confined to the rostrocaudal axis. Across all subjects, we observed the greatest migration when comparing the intraoperative fluoroscopy image with the x-ray from the first week (Figure 2, Supplementary Table S1). Relatively minimal rostrocaudal migration occurred in subsequent weeks for all subjects except for Subject 2. In the mediolateral axis, migration was more variable for both initial and total migration (Figure 3, Supplementary Table S2).

Across all leads for Subject 1 (Figure 3), the initial (i.e., from implant to Week 1) and total (i.e., from implant to explant) average rostrocaudal lead migration were 27.3 mm (SD: 9.3 mm) caudally and 29.7 mm (SD: 10.2 mm) caudally, respectively. In Subject 1, 8-contact SCS leads were utilized, and leads were inserted up to the C5 level. Given the significant amount of lead migration in Subject 1, we transitioned to 16-contact leads in Subject 2 and attempted to insert them further rostral (to C4) so that we could mitigate any caudal migration by adjusting which electrodes were used for stimulation. In theory, longer leads with more contacts, placed more rostrally, should experience greater frictional forces within the epidural space given that a larger surface area of the lead resides within the epidural space, thus offering greater lead stability. In that subject, two of the three leads maintained greater stability within the epidural space, with the exception of the most caudally inserted lead, which entered the epidural space at the T2-T3 interspace. Across all leads for Subject 2, the initial and total average rostrocaudal lead migration was 32.9 mm (SD: 34.5 mm) caudally and 41.6 mm (SD: 36.0 mm) caudally, respectively. For the two leads that did not migrate out of epidural space, the initial and total rostrocaudal lead migration was 13.1 mm (SD: 4.8 mm) caudally and 21.3 mm (SD: 10.5 mm) caudally, respectively. Because of ongoing personal matters, Subject 2 opted for removal of their leads explanted after Week 2 x-rays were collected.

Because we observed the most substantial lead migration in the first two subjects when electrodes were inserted at the T2-T3 interlaminar space, in Subjects 3 and 4 we decided to continue using 16-contact leads but to insert them superiorly (i.e., at C7-T1 or T1-T2 interlaminar space). For Subject 3, the initial and total rostrocaudal lead migration were 3.8 mm (SD: 1.5 mm) caudally and 1.9 mm (SD 2.9 mm) caudally, respectively. For Subject 4, the initial and total rostrocaudal lead migration were 18.7 mm (SD: 4.9 mm) caudally and 16.6 mm (SD: 1.8 mm) caudally, respectively. This combination of rostrally-placed 16-contact leads and more superior entry into the epidural space may have contributed to decreased initial rostrocaudal migration in comparison to Subjects 1 and 2 and similar minimal rostrocaudal migration in subsequent weeks. While for Subjects 1 and 2 there was overall continued caudal migration in all leads between Week 1 and explant, for Subjects 3 and 4, after initial caudal migration in the first week after implantation across all leads, there was slight rostral migration in two out of three leads in each subject and only minimal caudal migration in the other lead (Supplementary Table S1).

**Figure 4:**
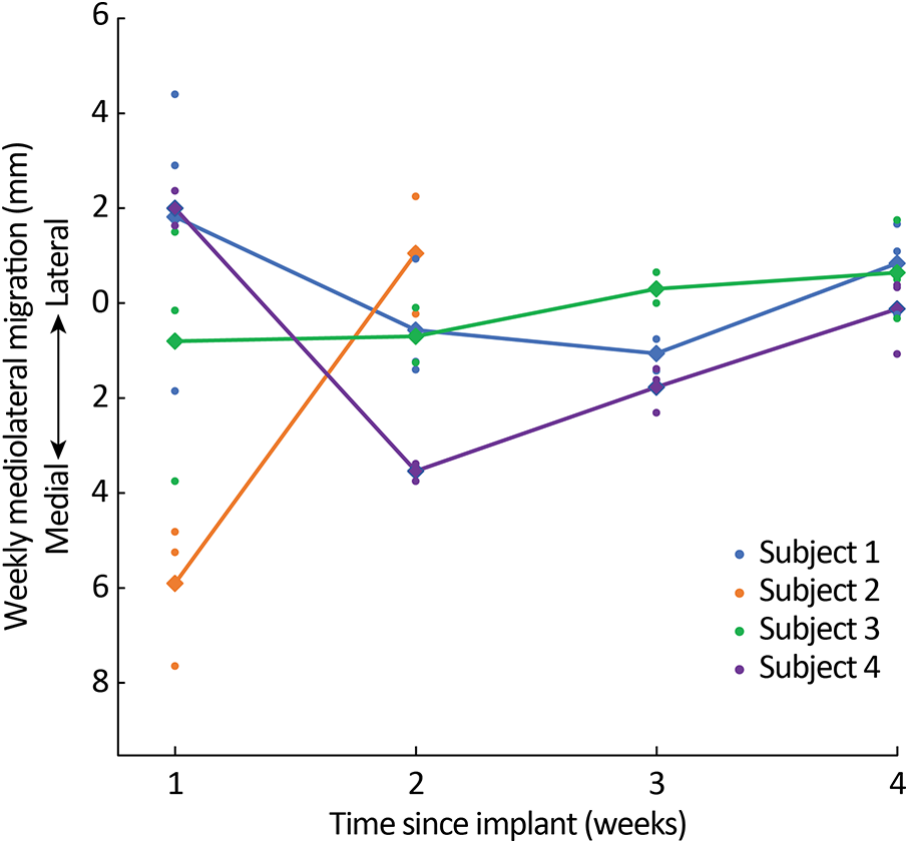
Mediolateral migration across weeks. Dots represent the migration of individual leads and diamonds/lines represent average migration across all leads for each subject.

When averaging across all leads per subject (Figure 4), the total mediolateral lead migration from implant to explant was 1.0 mm (SD: 3.4 mm) laterally in Subject 1, 4.9 mm (SD: 2.6 mm) medially in Subject 2, 0.6 mm (SD: 3.2 mm) medially in Subject 3, and 3.4 mm (SD: 0.6 mm) medially in Subject 4 (Supplementary Table S2).

**Figure 5:**
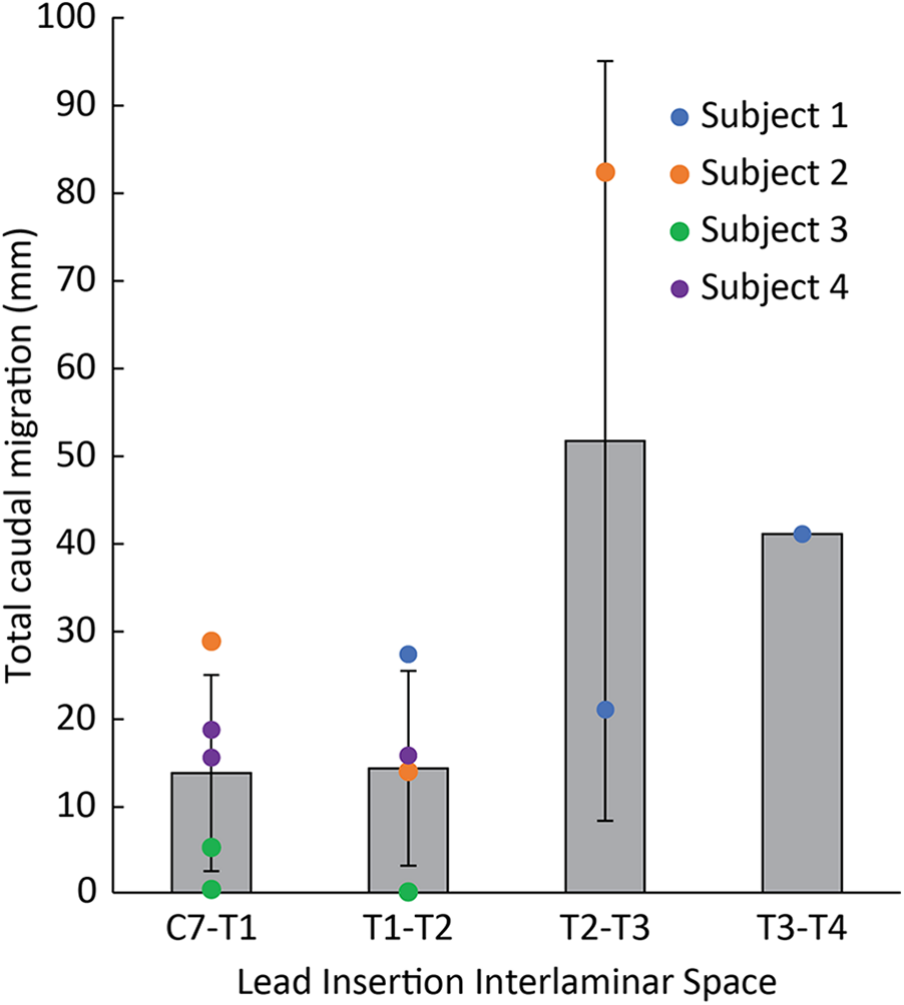
Total rostrocaudal lead migration from implant until explant across all leads based on lead insertion site. Individual lead migration indicated by circles. Average lead migration indicated by gray bars.

When assessing lead migration across subjects based on the location of the insertion site (Figure 5), leads inserted at a more rostral interlaminar space had decreased rostrocaudal migration. Across all leads in all subjects, the average total rostrocaudal migration was 13.7 mm (SD: 11.2 mm, 5 electrodes) caudally for leads placed at C7-T1, 14.2 mm (SD: 11.1 mm, 4 electrodes) caudally for T1-T2, 51.5 mm (SD: 43.4 mm; 2 electrodes) caudally for T2-T3, and 40.9 mm (1 electrode) caudally for T3-T4.

## 4 Discussion

Throughout this study, our iterative lead implantation strategy (i.e., using 16-contact leads, implanted more rostrally via a more rostral insertion site) demonstrated a reduction in the initial and total rostrocaudal lead migration. Similarly, there was a progressive reduction in total rostrocaudal lead migration for leads placed more rostrally regardless of subject. After the first week of lead placement, the mean rostrocaudal migration across the three leads for Subjects 1, 3, and 4 did not exceed 3 mm for the remainder of the 4-week trial period. Subject 2 requested lead explanation after the second week, so it is not possible to make a similar characterization. Average mediolateral lead migration was less than 5 mm throughout the duration of the study for all subjects.

The site of lead insertion seems to play an important role in limiting lead migration. In Subject 1, leads were placed in the most inferior interlaminar entry sites (i.e., T2-T3 and T3-T4), and these leads saw the most rostrocaudal migration as illustrated in Figure 5. In Subject 3, who experienced the smallest amount of migration, leads were placed in the most superior interlaminar entry sites, with leads entering the epidural space at the C7-T1 or T1-T2 interlaminar space. Subject 4 had leads similarly entering at the C7-T1 or T1-T2 interlaminar sites, and, while there was more initial rostrocaudal migration during the first week postoperatively in comparison to Subject 3, none of the leads migrated as much as the lead inserted at T2-T3 in Subject 2. We believe that migration phenomenon is likely related to scapular biomechanics. When the scapula retracts and elevates, these motions are largely mediated by the rhomboid minor and rhomboid major muscles, which originate at the spinous processes of C7 to T5 vertebrae and insert onto the medial scapular border. It is possible that during scapular retraction or elevation, shortening of these rhomboid fibers generates a substantial force at the middle of their origin (namely, at the T2-T3 spinous processes). This might explain why leads inserted at the C7-T1 and T1-T2 interspace exhibited minimal caudal migration, whereas the leads inserted at T2-T3 and T3-T4 exhibited more substantial migration. While both Subjects 3 and 4 had similar rostral lead insertion sites, differences in migration could have occurred because of greater forces at the C7-T1 origins of the rhomboid major and minor muscles in Subject 4, who had a lower level of amputation and therefore a longer lever arm while the scapula retracted/stabilized.

In our cohort, we obtained serial x-rays to evaluate lead placement and compared them to intraoperative fluoroscopic images, which to our knowledge has not occurred in any prior study. This allowed us to calculate the true lead migration rate over the 29-day course of the study, which was negligible when entering the interlaminar space at the C7-T1 level. This observation is even more compelling when considering our method of securing the percutaneous SCS leads, which involved suturing lead anchors into superficial tissue near the lead exit site. In clinical practice, most permanent SCS lead implants are reinforced with muscle fascia anchors and/or strain relief loops, which typically result in less lead migration than our method of cutaneous tissue anchoring (19). Perhaps more superior lead entry for cervical SCS, in conjunction with muscle fascia anchoring and strain relief loop formation, would further augment lead stability and reduce overall procedural cost due to decreased need for lead revisions.It is important to note, however, several limitations when interpreting the results of this study. This was a small cohort case series and the approach to lead placement was iteratively modified between procedures. In the future, a larger sized cohort study should be performed to confirm the effects of these techniques on lead migration. Additionally, the intraoperative fluoroscopic images were collected in a prone position, while all subsequent x-rays were collected in standing. Care was taken to achieve a similar posture during x-rays, although some differences inevitably occurred and make affect the results reported here. In our analysis, we used multiple bony landmarks to align x-rays when measuring lead migration, though slight changes in position may contribute to errors in those measurements, especially with respect to mediolateral migration. Finally, multiple days occurred between collection of intraoperative fluoroscopy and x-rays, limiting our ability to understand the degree to which migration occurred immediately after the subject stood up vs. in the week following explant.

## 5 Conclusions

Regardless of these limitations, the results of this study suggest that utilizing rostrally-placed 16-contact SCS leads and entering the epidural space at superior interlaminar levels (specifically the C7-T1 level) results in decreased lead migration, which may prove useful for clinicians looking to mitigate this common post-implantation complication and improve cervical SCS efficacy.

In our small cohort, percutaneous placement of lateral cervical SCS leads resulted in minimal rostrocaudal migration when utilizing 16-contact SCS leads, when placing leads as rostrally as possible, and when entering superior interlaminar spaces (with the least amount of migration observed when entering the C7-T1 interlaminar space).

## Data Availability

The original contributions presented in the study are included in the article/Supplementary Material, further inquiries can be directed to the corresponding authors.
