# Supplementary material for: Techniques to mitigate lead migration for percutaneous trials of cervical spinal cord stimulation
Source: Front Surg. 2025 Mar 31;12:1458572. doi: 10.3389/fsurg.2025.1458572 (PMC11994673; doi:10.3389/fsurg.2025.1458572)
Supplement: Supplementary file 1 [file Datasheet1.pdf]

Supplementary Tables

Table S1. Rostrocaudal migration (scaled measurements in mm). Negative denotes caudal migration, positive denotes rostral migration.

		Implant to Day 7	Day 7 to 14	Day 14 to 21	Day 21 to 28	Implant to Day 28	Day 7 to 28	Average weekly migration from implant to removal	Average weekly migration Day 7 to removal
Subject 1									
Individual leads	1	-37.3	-4.0	4.0	-3.7	-40.9	-3.7	-10.2	-1.2
	2	-18.7	-4.2	6.1	-4.1	-20.9	-2.2	-5.2	-0.7
	3	-26.1	1.8	2.3	-5.3	-27.3	-1.2	-6.8	-0.4
Average		-27.3	-2.1	4.1	-4.4	-29.7	-2.4		
Subject 2									
Individual leads	1	-9.7	-4.1			-13.8	-4.1	-6.9	-4.1
	2	-72.5	-9.7			-82.2	-9.7	-41.1	-9.7
	3	-16.6	-12.2			-28.7	-12.2	-14.4	-12.2
Average		-32.9	-8.7			-41.6	-8.7		
Subject 3									
Individual leads	1	-2.0	2.5	2.1	-3.0	-0.4	1.6	-0.1	0.5
	2	-5.0	4.0	0.7	0.2	-0.1	4.8	0.0	1.6
	3	-4.3	0.5	-0.7	-0.8	-5.2	-0.9	-1.3	-0.3
Average		-3.8	2.3	0.7	-1.2	-1.9	1.8		
Subject 4									
Individual leads	1	-22.7	4.0	1.4	1.6	-15.7	7.0	-3.9	2.3
	2	-20.3	-0.3	1.2	0.8	-18.6	1.7	-4.7	0.6
	3	-13.2	-2.9	2.3	-1.6	-15.5	-2.3	-3.9	-0.8
Average		-18.7	0.3	1.6	0.3	-16.6	2.1		

Table S2. Medial-lateral weekly migration (scaled measurements in mm). Negative denotes medial migration, positive denotes lateral migration.

		Implant to Day 7	Day 7 to 14	Day 14 to 21	Day 21 to 28	Implant to Day 28	Day 7 to 28	Average weekly migration from implant to removal	Average weekly migration Day 7 to removal
Subject 1									
Individual leads	1	-1.9	-1.2	-0.8	1.1	-2.8	2.3	-0.7	-0.3
	2	4.4	-1.4	-1.0	1.7	3.7	3.1	0.9	-0.2
	3	2.9	0.9	-1.4	-0.2	2.2	-1.2	0.5	-0.2
Average		1.8	-0.6	-1.1	0.8	1.0	1.4		
Subject 2									
Individual leads	1	-5.3	2.3			-3.0	2.3	-1.5	2.3
	2	-7.6	-0.2			-7.9	-0.2	-3.9	-0.2
	3	-4.8	1.1			-3.7	1.1	-1.8	1.1
Average		-5.9	1.1			-4.9	1.1		
Subject 3									
Individual leads	1	-3.8	-0.8	0.0	0.5	-4.0	-0.3	-1.0	-0.1
	2	1.5	-1.3	0.3	1.8	2.3	0.8	0.6	0.3
	3	-0.2	-0.1	0.7	-0.3	0.1	0.2	0.0	0.1
Average		-0.8	-0.7	0.3	0.6	-0.6	0.2		
Subject 4									
Individual leads	1	1.6	-3.5	-1.4	0.3	-2.9	-4.5	-0.7	-1.5
	2	2.0	-3.4	-1.6	-1.1	-4.1	-6.1	-1.0	-2.0
	3	2.4	-3.8	-2.3	0.4	-3.3	-5.7	-0.8	-1.9
Average		2.0	-3.5	-1.8	-0.1	-3.4	-5.4		

Supplementary Figures

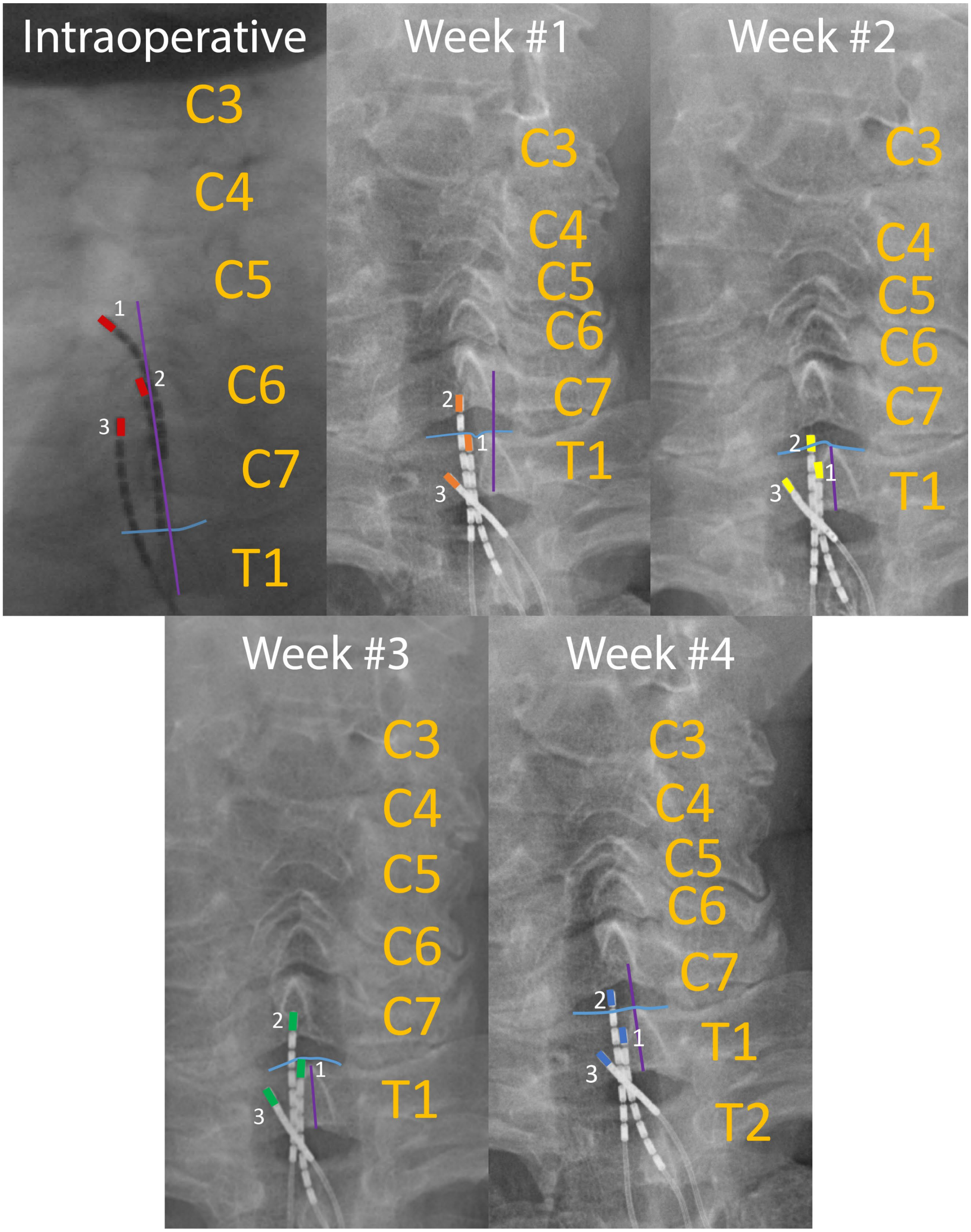

Figure S1. Annotated intraoperative fluoroscopic image and weekly x-rays for Subject 1.

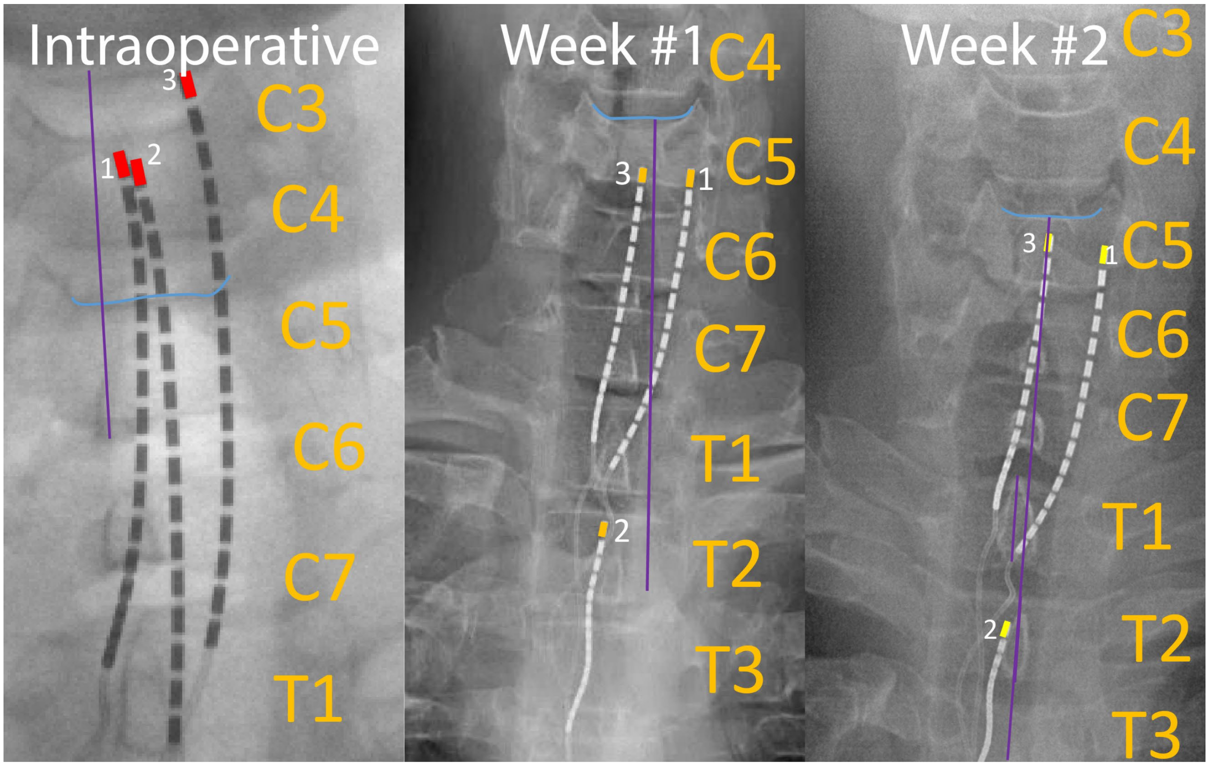

Figure S2. Annotated intraoperative fluoroscopic image and weekly x-rays for Subject 2.

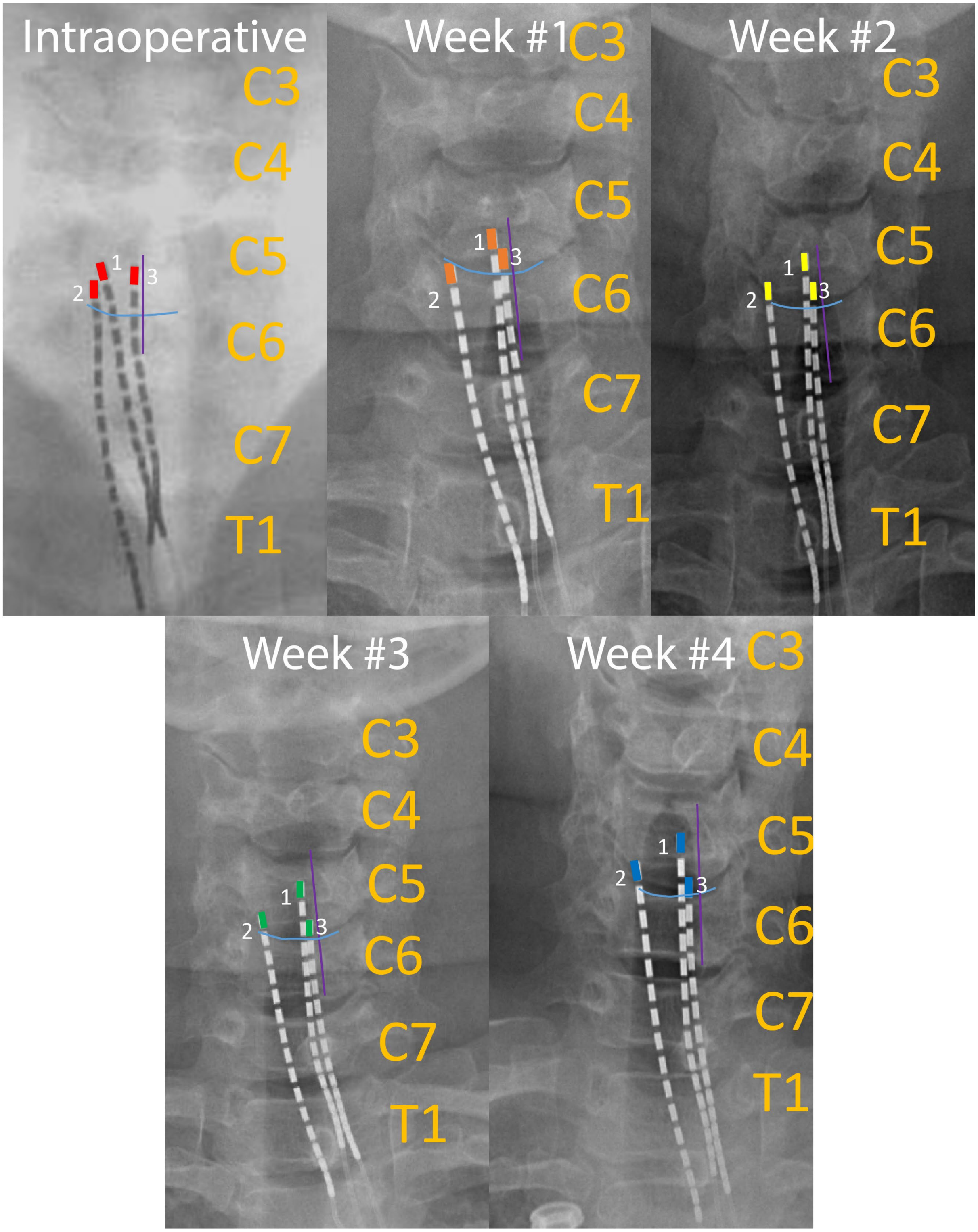

Figure S3. Annotated intraoperative fluoroscopic image and weekly x-rays for Subject 3.

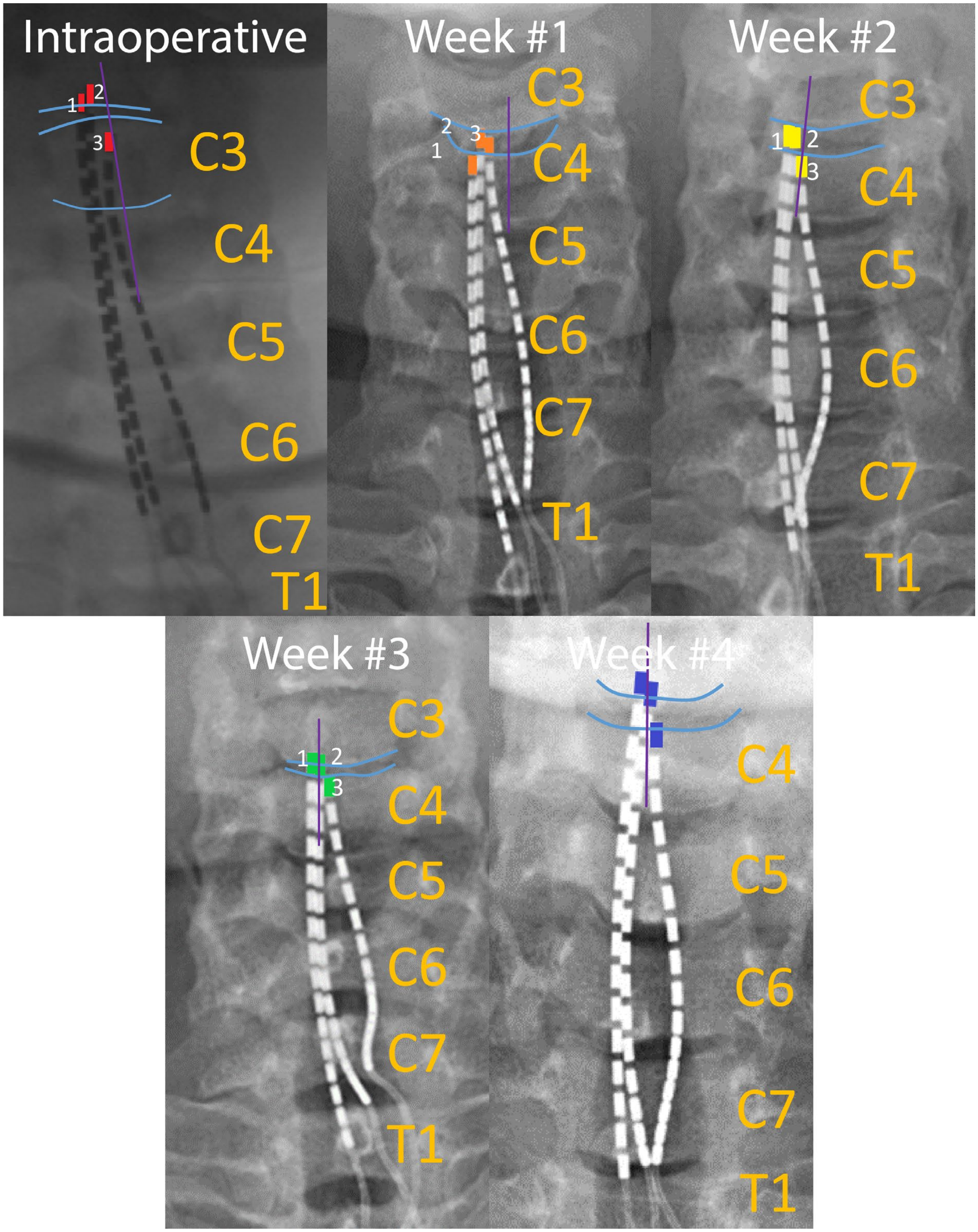

Figure S4. Annotated intraoperative fluoroscopic image and weekly x-rays for Subject 4.
